# Recency is sufficient for reconciling categorisation and memory: Commentary on Devraj et al. (2024)

**DOI:** 10.3758/s13423-025-02780-9

**Published:** 2026-01-15

**Authors:** Daniel R. Hutchinson, Daniel R. Little, Adam F. Osth

**Affiliations:** Complex Human Data Hub, University of Melbourne, Level 8 Melbourne Connect, 700 Swanston St, Carlton, VIC 3053 Australia

**Keywords:** Category learning, Exemplar models, Prototype models, Forgetting, Memory retrieval

## Abstract

Devraj et al. (*Psychonomic Bulletin and Review*. https://doi.org/10.3758/s13423-023-02448-2,  2024) argued that findings which suggest that memories for items become less accessible over time conflict with categorisation findings where exemplar performance improves across training. To reconcile this, they highlighted that under real-world conditions items tend to reappear less frequently over time, thus preferentially maintaining new items can improve performance. Typical categorisation experiments instead distribute exemplars uniformly across trials. However, under a power-law stimulus distribution, Devraj et al. showed worsening fit for exemplar classification models across trials. They used this as evidence that forgetting behaviour adapted to task demands, reducing exemplar accessibility and encouraging prototype use for classification decisions. By re-analysing the same data, we argue instead that this pattern can be produced with exemplar-forgetting in both conditions. By systematically increasing in the delays across which stimuli were tested, their Experimental condition exaggerated the effects of forgetting on performance in later trials compared to the Control condition. This resulted in a reversal of performance growth across trials – instead leading to a steady decline in performance. As exemplar model-fit advantage is expected to vary with performance, we suggest that trends in this advantage are not diagnostic of a shift in classification strategy. We found that a forgetting-function improved exemplar model fit to Devraj et al.’s data, and under reasonable parameters could predict the observed patterns of performance and model-fit a priori. Compared with a strategy-shifting mixture model, exemplar-forgetting provided equivalent fits despite being more theoretically parsimonious. We suggest power-law memory decay does not produce a tension between categorisation and memory findings, as increased forgetting is found across longer retention intervals, whereas the delay between exemplar learning and classification remains constant across typical categorisation experiments.

**Supplementary Information:**

The online version contains supplementary material available at 10.3758/s13423-025-02780-9.

## Introduction

Theories of categorisation agree that items are classified according to their similarity to category representations but disagree about the structure of these representations. Prototype models assume that a single ‘ideal member’ for each category is stored, often formed from an average of other members (Posner & Keele, 1968), whereas exemplar models (Hintzman, 1986; Nosofsky, 1986) propose exemplars are stored individually. While well-learned categories are typically best fit by exemplar models (Nosofsky & Zaki, 2002; Smith & Minda, 2000), Smith and Minda (2001, 2000, 1998) argued that early in category learning, performance profiles suggest prototype use, and prototype models show an advantage. However, subsequent work has demonstrated that exemplar representations alone are sufficient to produce this pattern^1^ (Nosofsky & Zaki, 2002).

Devraj et al. (2024) reasoned that the early-in-learning prototype model advantage demonstrated by Smith and Minda (1998, 2000) are at odds with findings suggesting that memory for individual exemplars degrades more than for prototypes over long retention intervals (Posner & Keele, 1970). Devraj et al. argued that experimental paradigms involving independent sampling of stimuli – of which Smith and Minda’s design is an example – are not representative of real-world memory conditions where recent exemplars are disproportionately likely to be re-experienced (Anderson & Schooler, 1991). If recent items re-appear more frequently, information about older items should become less valuable over time. Categorization experiments that instead use the more typical uniform independent sampling regime may lead participants to cease prioritisation of recent information since all items are equally likely to reoccur.

**Fig. 1:**
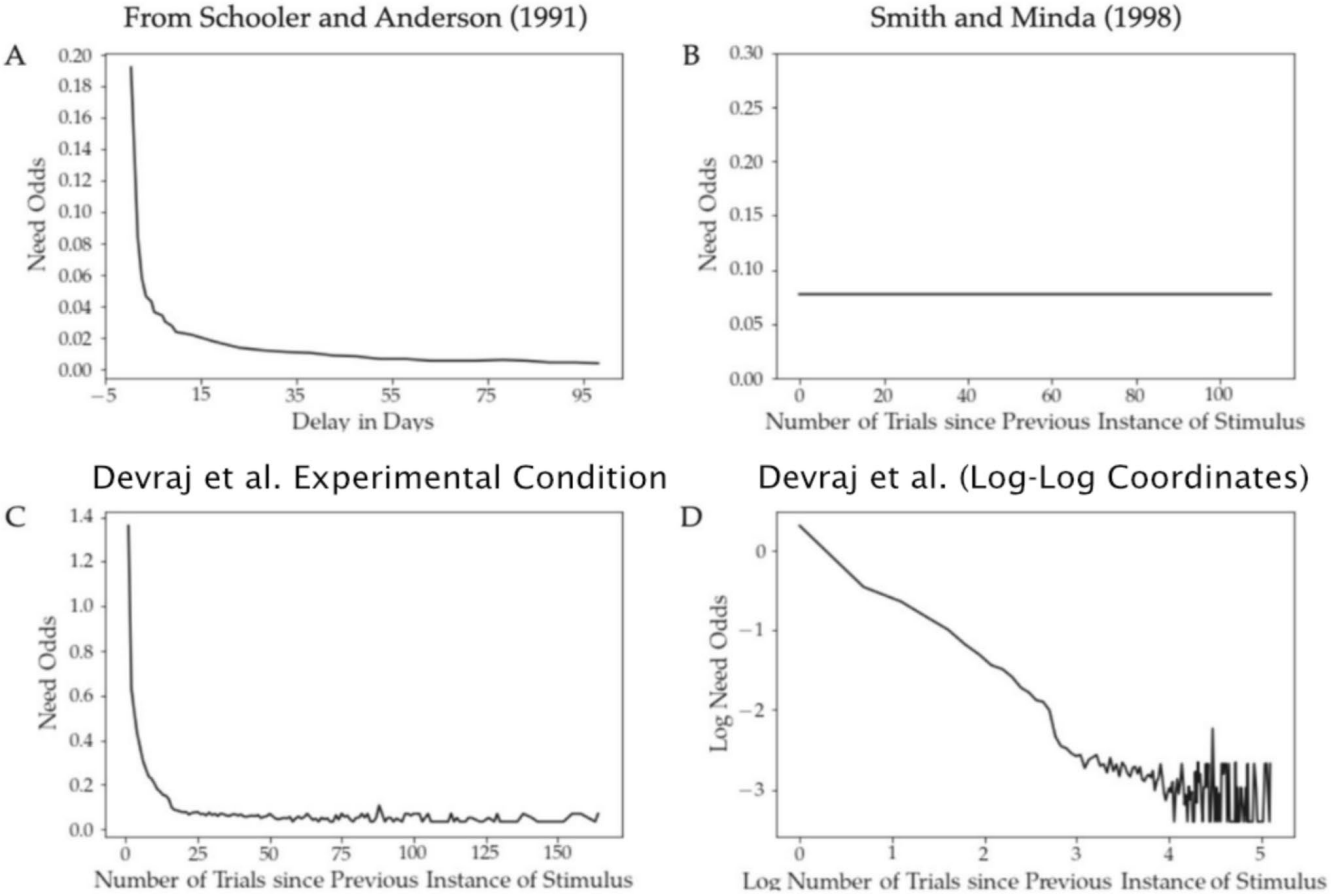
Environmental recency functions, displaying the relationship between stimulus recency and its need odds, calculated as P(stim)/(1− P(stim)), where P(stim) is the probability of seeing the stimulus. In newspaper headlines (**A**) from Anderson (1991), in Smith and Minda’s (1998) experimental design (**B**), and in Devraj et al. (2024) (**C, D**). Adapted from *“Reconciling categorization and memory via environmental statistics,”* by A. Devraj, T. L. Griffiths, and Q. Zhang, 2024*, Psychonomic Bulletin & Review, (*10.3758/s13423-023-02448-2*).* Copyright 2024 by Springer Nature. Reprinted with permission

To investigate this hypothesis, Devraj et al. performed a categorisation experiment in which stimuli were presented according to a power-law distribution such that the presentation frequency of a stimulus decreased over time, and the average delay since it was last seen increased, to approximate the environmental statistics of real-world category learning. Figure 1 shows how the environmental recency in Devraj et al.’s power-law condition (Fig. 1C) approximates the real-world recency estimates from Schooler and Anderson (Fig. 1A) and contrasts with typical experimental recency (Fig. 1B). In other words, while in the Smith and Minda design (and in most typical categorization experiments) all stimuli were always equally likely to appear, in Devraj et al.’s power-law condition, newly or recently presented stimuli were more likely to re-appear sooner and older stimuli were more likely to either re-appear after a long delay or not at all.

**Fig. 2:**
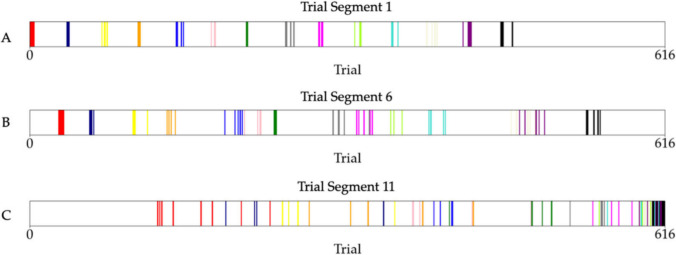
Lines of the same colour represent trials where the same stimulus was displayed. In later segments, appearances of the same stimuli are less frequent. From *“Reconciling categorization and memory via environmental statistics,”* by A. Devraj, T. L. Griffiths, and Q. Zhang, 2024*, Psychonomic Bulletin & Review, (*10.3758/s13423-023-02448-2*).* Copyright 2024 by Springer Nature. Reprinted with permission

In Devraj et al., participants classified 14 nonsense words into two categories with a rule-plus-exception structure. Words from the same category shared similar letters, except for a single ‘exception’ word, which shared all but one letter with the *alternate* category. In the Control condition, stimuli were uniformly distributed across trials. In the Experimental condition, after first presentation, a stimulus would re-appear very frequently but became rarer across trials. Control condition trials were binned sequentially into ‘segments’ for analysis, but Experimental condition segments represented trials with equivalent stimulus frequencies (see Fig. 2). For clarity, we will refer to Control segments as *sequential segments* (as they are temporally contiguous) and Experimental segments as *power-law segments* (which are discontiguous).

In the Experimental condition, classification accuracy was highest for trials in early power-law segments, where stimuli repeated frequently, but diminished across segments. In the Control condition, accuracy gradually increased across trials. Similarly, the model-fit advantage for exemplar models was largest early in power-law segments, but in the Control condition, the exemplar advantage steadily increased over time. Devraj et al. interpreted these trends as evidence that the accessibility of exemplar-specific memories depends on their relevance in future decisions. Under the power-law, when older exemplars become less relevant over time, forgetting them in favour of new ones becomes adaptive. Devraj et al. assumed that this leads to an increased reliance on prototype information when old, forgotten exemplars are occasionally tested. By contrast, under uniform presentation, exemplars remain equally relevant over time with no adaptive benefit for forgetting. As a result, exemplars are maintained and ‘overlearned’ across repeated presentations, increasing accuracy over time.

**Fig. 3:**
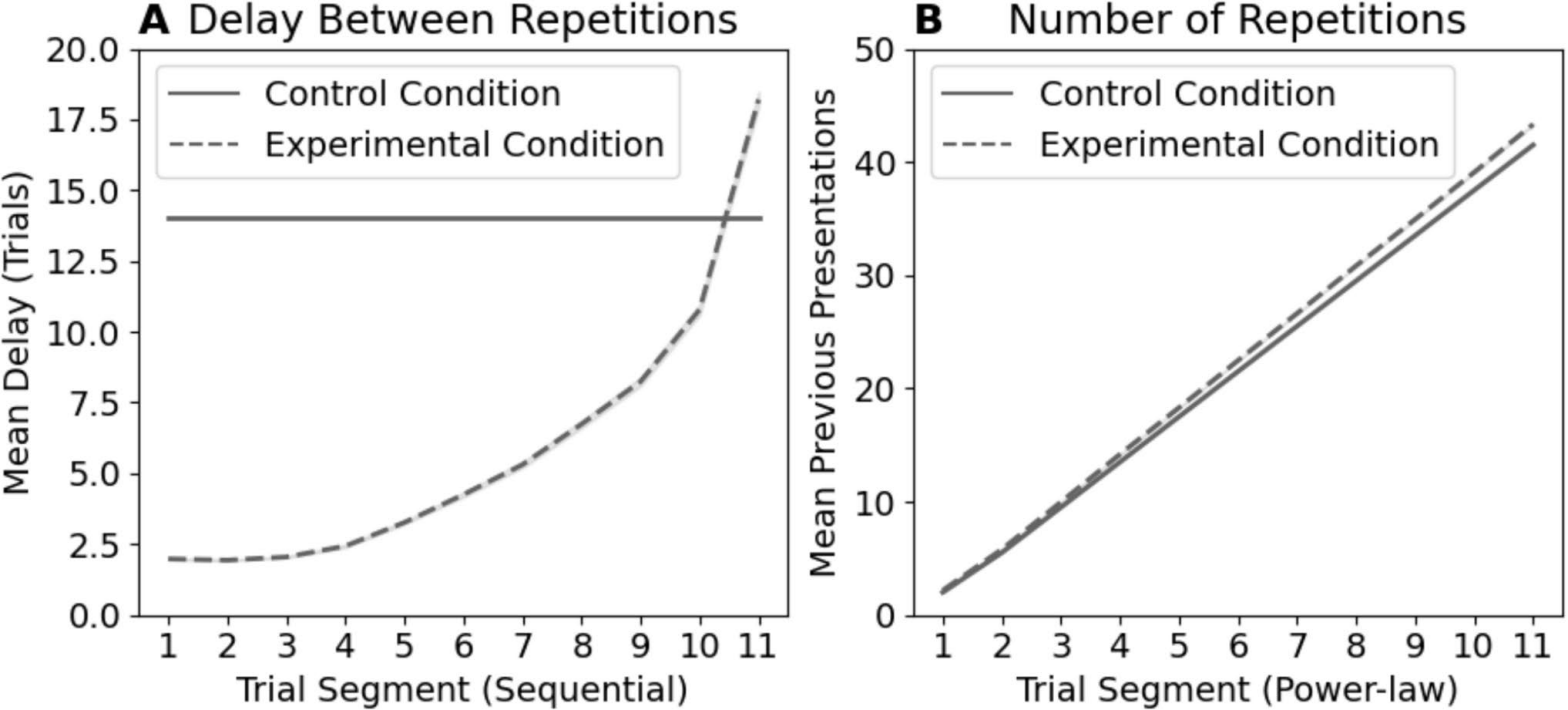
Panel **A** shows the mean delay in trials between repeated presentations of the same stimulus per *(sequential)* segment. Note that later power-law segments show longer delays between repetitions (reduced recency). Panel **B** shows instead the average number of times any tested stimulus has previously been encountered at each *(power-law)* segment. Note that this is roughly equivalent between conditions

Rather than reflecting adaptive shifts in relative exemplar and prototype representation accessibility between conditions, we instead argue that the seemingly opposing patterns of performance and model-fit in both conditions are consistent with an exemplar-based representation in which memories for exemplars decay with a constant power-law forgetting rate. Consider two simple premises: repeated presentations of exemplars improve discrimination, and exemplar memories decay according to a power-law over time. In the Control condition, exemplar recency – the delay since last seeing a stimulus – varied across a small range and was constant on average across sequential segments (as in Fig. 3A). In the Experimental condition, however, stimuli are initially experienced in ‘bursts’ of frequent repetitions, followed by increasingly long intervening periods of other stimuli (Fig. 3A). In other words, recency varies considerably between trials and systematically decreases across power-law segments. When recency is constant across sequential segments, as in the Control condition, the effect of forgetting is also constant and repeated presentations drive learning and increase performance (Fig. 3B). However, when recency *systematically decreases* across power-law segments in the Experimental condition, forgetting also increases and dominates any improvements due to learning, resulting in worsened performance over time.

We begin by discussing why relative model-fit for prototype and exemplar models changes across segments in this experiment and argue that these shifts do not necessitate differences in underlying category representations. We then demonstrate that an exemplar model with forgetting provides improved fits to data over both the standard exemplar and the prototype models. Data simulated from this model demonstrated the distinct patterns of accuracy across conditions a priori under identical parameters. Importantly, this included forgetting rate, suggesting that no difference in exemplar accessibility over time across conditions – and thus no adaptation to environmental recency – is necessary to produce this effect*.* We additionally fit this simulated data with both the base exemplar model and the prototype model showing the same pattern of changes in relative model fit as Devraj et al. to reinforce that the relative changes in model-fit cannot be used to diagnose a strategic change in representation use. We argue that systematic differences in environmental recency should be *expected* to produce different patterns of performance even if only exemplar-based representations are used. Finally, using a model that mixes prototype and exemplar representations, we demonstrate that both explanations are equally capable of capturing the data, but a forgetting explanation is more consistent with previous findings and more parsimonious.

## Method

### Stimuli

Stimuli were 14 six-letter nonsense-words with alternating consonants and vowels. Each letter could take on one of two values, shared with either the category A or the category B prototype. For example, the category A prototype could be ‘gafuzi’, and the category B prototype ‘livamo’, coded as 000000 and 111111, respectively. Five additional category A stimuli are created by swapping one letter with the other prototype (e.g., ‘gifuzi’; 010000). Finally, an exception item was created for category A by changing one value of the category B prototype (e.g., ‘livazo’; 111101). Category B stimuli follow the same pattern. For modelling, each letter represents one feature which can either match or mismatch when comparing two words.

### Design

On each of 616 trials, participants categorised a stimulus and then received feedback (i.e., *Correct* or *Incorrect*). In the Control condition, every 14 trials were a random permutation of all stimuli. In the Experimental condition, by contrast, new stimuli were introduced every 35 trials then reappeared in accordance with a power-law, initially frequent but rarer over time.

For analysis, Devraj et al. binned trials into 11 segments, representing blocks of 56 sequential trials in the Control condition. In the Experimental condition, segments contained trials from similar points along the power-law curve. The first segment contained the initial few appearances of all 14 stimuli, each recently introduced and repeating frequently. As stimuli were introduced one by one, trials in this segment were distributed temporally throughout the experiment (Fig. 2). Later segments represent longer delays between stimulus repetitions (see Fig. 3A).

### Computational modelling

In the prototype model, observed stimuli are classified based on comparisons with each category prototype. First, the distance between stimulus *i* and Prototype A is calculated as:

1 $${d}_{\mathrm{iA}}=\sum {w}_{\mathrm{m}}\left|{x}_{\mathrm{im}}-{P}_{\mathrm{Am}}\right|,$$

where ${x}_{\mathrm{im}}$ is the value of item *i* on dimension *m*, ${P}_{\mathrm{Am}}$ is value of dimension m for Prototype A. Attention weights, ${w}_{\mathrm{m}}$, lie between 0 and 1, and sum to 1. Similarity of item *i* to Prototype A is computed from its distances by:

2 $${\mathrm{s}}_{\mathrm{iA}}=\mathrm{exp}\left(-c{d}_{\mathrm{iA}}\right),$$

where *c* is a sensitivity parameter. The similarity to Prototype B is calculated analogously. Finally, the probability of stimulus *i* being classified into category A is given by:

3 $$P\left(\mathrm{A}|i\right)=\frac{{\mathrm{s}}_{\mathrm{iA}}}{{\mathrm{s}}_{\mathrm{iA}}+{\mathrm{s}}_{\mathrm{iB}}}.$$

Under the exemplar model, observed stimuli are instead compared to all stored exemplars from both categories. First, the distances between two exemplars *i* and *j* is given by:

4 $${d}_{\mathrm{ij}}=\sum_{m=1}^{M}{w}_{\mathrm{m}}\left|{x}_{\mathrm{im}}-{x}_{\mathrm{jm}}\right|,$$

where ${x}_{\mathrm{im}}$ and ${x}_{\mathrm{jm}}$ are the values of exemplars *i* and *j* on dimension *m*, respectively. Attention weights, ${w}_{\mathrm{m}}$ lie between 0 and 1, and sum to 1. The similarity of any two exemplars *i* and *j* are computed from their distance by:

5 $${\mathrm{s}}_{\mathrm{ij}}=\mathrm{exp}\left(-c{d}_{\mathrm{ij}}\right).$$

Finally, the probability of stimulus *i* being classified into category A is given by:

6 $$\mathrm{P}\left(\mathrm{A}|i\right)=\frac{\sum {s}_{\mathrm{ia}}}{\sum {s}_{\mathrm{ia}} + \sum {s}_{\mathrm{ib}}},$$

where $\sum {s}_{\mathrm{ia}}$ and $\sum {s}_{\mathrm{ib}}$ are the summed similarities of exemplar *i* to all exemplars from category A and B, respectively.

We utilised the same model formulations to remain consistent with Devraj et al. (2024), where both models have six free parameters each (six attention weights with five free given the sum-to-one constraint, and one sensitivity parameter). Consequently, we did not include a response-scaling mechanism for the exemplar model. While response-scaling is crucial for exemplar models to explain early category-learning behaviour in similar tasks (see Nososfky & Zaki, 2002), we believe that including a forgetting mechanism is key for understanding an explicit recency manipulation. By omitting response-scaling, we simplify comparisons between our exemplar-forgetting model (described in Analysis 2) and the exemplar model discussed by Devraj et al. (2024).

### Model fitting

Our model-fitting procedure was mostly a direct replication of Devraj et al. (2024). In the Control condition, their models were fit to each sequential segment for each participant separately. They used Sequential Least Squares Programming (SLSQP) to minimise sum-squared error (SSE), calculated as:

7 $$SSE=\sum_{i=1}^{14}{\left[P\left({R}_{\mathrm{A}}|{S}_{i}\right)-{\widehat{p}}_{\mathrm{A}, {s}_{i}}\right]}^{2},$$

where ${\widehat{p}}_{\mathrm{A}, {s}_{i}}$ is the proportion of observed category A responses for stimulus *i* across the segment and $P\left({R}_{\mathrm{A}}|{S}_{i}\right)$ the model’s predicted proportion of A responses for stimulus *i*. In their Experimental condition, sequential trials can be binned into different power-law segments. Devraj et al. deemed that changing model parameters in response to trial-level frequency information was psychologically implausible, so models were fit to SSE per participant for all trials simultaneously (as opposed to each power-law segment separately).

Although fit using SSE to all power-law segments simultaneously, the Experimental condition was *plotted* using MSE (mean-squared error) because experimental segments were *on average* 56 trials long, but segment length varied considerably between segments. Unlike SSE, which compared response proportions, MSE was calculated per trial:

8 $$MSE=\frac{1}{n}\sum_{j=1}^{n}{\left[P\left({R}_{\mathrm{A}}|{S}_{j}\right)-{R}_{\mathrm{A}j}\right]}^{2},$$

where n is the number of trials in the segment, and ${R}_{\mathrm{A}j}=1$ if the response on trial *j* was category A, and 0 otherwise. $P\left({R}_{\mathrm{A}}|{S}_{j}\right)$ is the model predicted probability of an A responses for stimulus *j*.

We closely replicated this method where possible to allow for simple comparisons between our analyses; however, we fit *both* conditions using MSE when testing whether exemplar-based forgetting could explain behaviour. Exemplar-forgetting suggests that classifications on *each trial* depend on the recency of exemplars from each category *on that trial*. This is not adequately tested with Devraj et al.’s SSE fitting method because they aggregated responses across each trial segment. As a result, a model that perfectly captures the effect of recency on trial-level performance may not show any advantage over one predicting *no* trial-level variation, as long as both predict the same *mean* response for a given stimulus across the segment. By contrast, their MSE statistic was calculated using error on each individual trial and can easily differentiate the two models. While under normal circumstances optimising for SSE and MSE would be equivalent, fits optimised to aggregated and individual-trial data are unlikely to be the same – particularly for the forgetting model. To avoid confusion here, we chose to always plot and fit models using the same fit statistic,^2^ using SSE/aggregated fits to allow direct comparison with Devraj et al. (2024) in the Control condition, but preferring MSE/trial-level fits in all other cases.

## Analysis 1: Performance on exception items

**Fig. 4:**
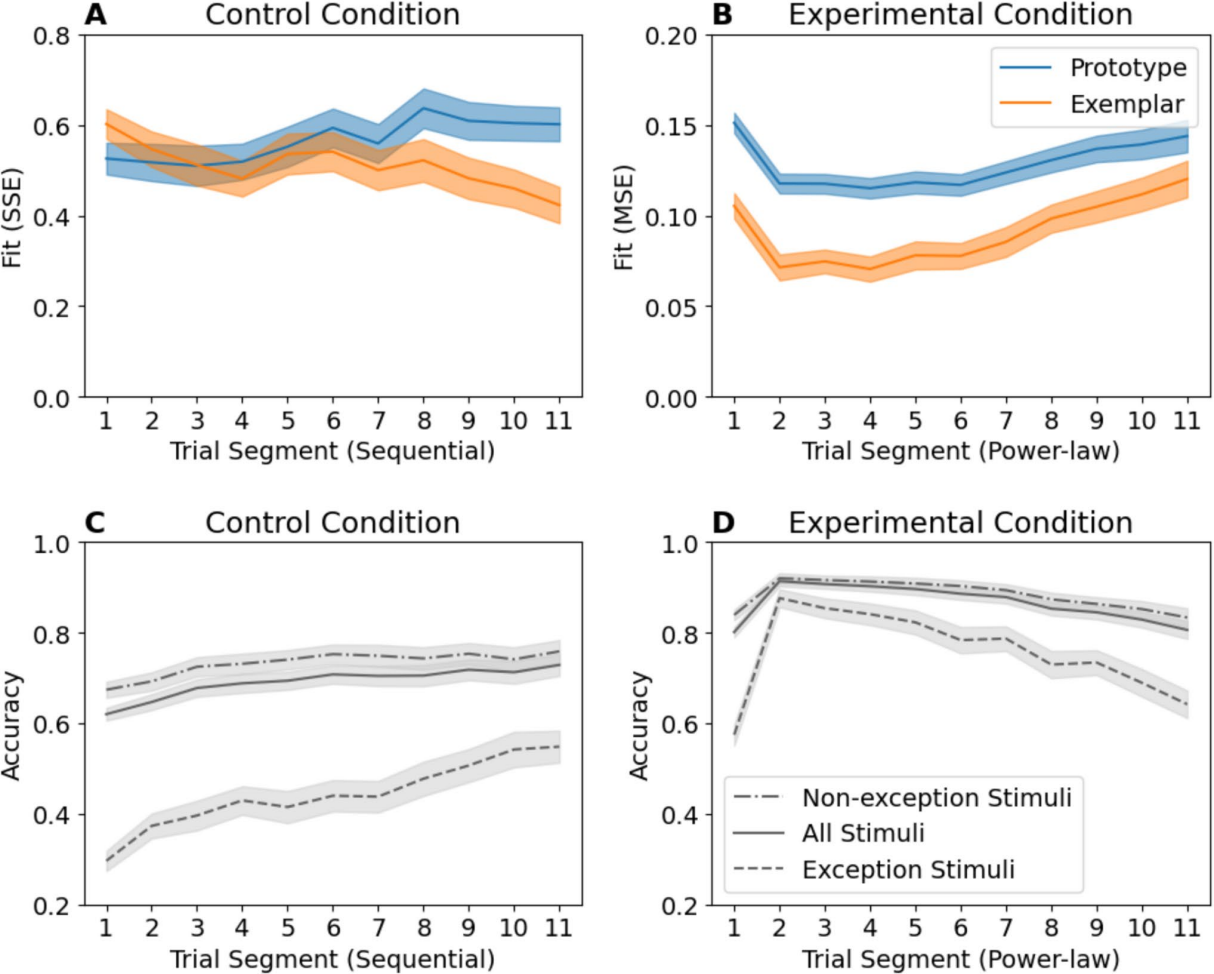
Replications of model fit plots from Devraj et al. (2024) Experiment 1 fit using sum-squared error (SSE) in the Control condition and mean-squared error (MSE) in the Experimental condition as described in *Methods* (**A, B**). Observed accuracy (**C**, **D**), but subdivided by item type. Error bars indicate standard error of the mean

The key evidence used to support the claim of a switch from reliance on exemplars to reliance on prototypes comes from examination of the difference in prototype and exemplar model fits across Trial Segments. In Fig. 4, for the Control condition, the exemplar model shows an initially higher SSE compared to the prototype model, whose fit got worse over sequential segments (Fig. 4A). For the Experimental condition, we instead see a large initial difference in MSE, which declines over time (Fig. 4B). The reason for the change in fit over time is exception item performance. Both models predict that items similar to the prototype for their category should be accurately classified. However, the prototype model predicts that exception items, which are more similar to the *alternate* category prototype, should *always* be poorly classified (Reed, 1972). The exemplar model instead predicts that exception performance depends on the sensitivity parameter *c,* which theoretically determines the ability to discriminate individual exemplars^3^ (Nosofsky, 1987; Smith & Minda, 1998). When exception accuracy *increases* across the Control condition (Fig. 4C), the exemplar model fit improves relative to the prototype model. When exception accuracy *declines* across the Experimental condition (Fig. 4D), so does the exemplar fit advantage.

**Fig. 5:**
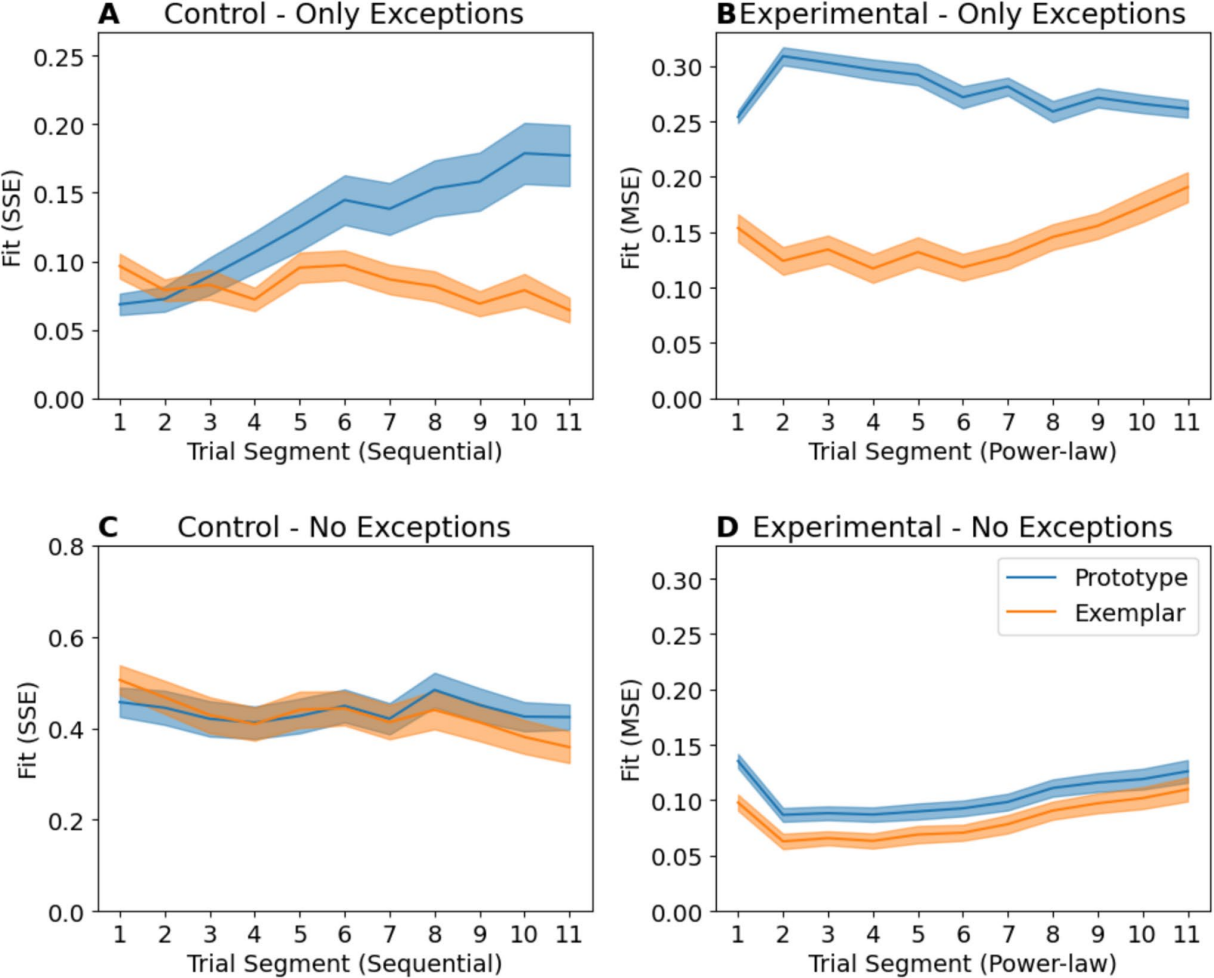
Comparison of model fits partitioned by item type. Note while Experimental condition panels (**B, D**) lie on the same scale and can be compared directly, Control condition panels (**A**,** C**) cannot. This is because sum-squared error (SSE) values depend on the number of stimuli being summed across, and there are two exceptions but 12 non-exceptions. Therefore, if exceptions are approximately equally well fit, their SSE in **A** should be one-sixth that of non-exceptions from the same model in **C**. Error bars indicate standard error of the mean

To demonstrate how impactful exceptions are on the model fit, we partition the model fit from Fig. 4 into the contributions of exception and non-exception items (Fig. 5). Exception items (Fig. 5A, B) disproportionately contribute to the *difference* in fit over time between prototype and exemplar models compared with non-exceptions (Fig. 5C, D).

To understand why this is important, consider that unlike exemplar-based models, prototype models cannot simultaneously predict accurate classification of both exceptions and non-exceptions. These models are primarily differentiated by their ability to predict exception item performance, and exemplar models will show a fit advantage proportional to exception item accuracy. Consequently, we should *expect* changes in the relative model fit if exception accuracy varies over time, even if participants always use exemplar-based classification.

This logic can also explain the findings of Devraj et al.’s Experiment 2, which found no exemplar model fit advantage over prototype models when exception items were removed from the design, and an increased advantage when additional exceptions were added. They argued that as exemplars are required for accurate exception classification, when exceptions are added, participants begin to utilise exemplar-based classification to maximise performance. With more exemplars, there is more incentive to maintain exemplar representations. We suggest an alternative explanation – these models are primarily differentiated by their predictions about exception items, and thus, without them, data are no longer diagnostic. Participants could be employing exemplar-based strategies in all three cases; however, this does not affect model-fits until exceptions are present. As more exceptions are added, even if identical strategies are employed, the model-fit advantage for exemplar models will grow. We next demonstrate that exemplar-forgetting can both capture and predict the observed patterns of performance-over-time.

## Analysis 2: Exemplar forgetting versus mixture of prototype and exemplar processing

While Devraj et al. suggest that the two conditions encourage different optimal patterns of forgetting and resultant classification strategy due to environmental adaptation, we instead argue that forgetting in both conditions always follows a power-law curve and can explain what appear to be distinct patterns of behaviour across their distributional manipulations. For both conditions we suggest that learning occurs through repeated exposure to stimuli, improving performance over time. Forgetting, in contrast, worsens performance relative to the delay since an item was last seen. Regardless of condition, stimuli in any given segment have been seen approximately the same number of times (see Fig. 3B), leading to approximately equivalent learning.^4^ In the Control condition, although the recency of the test stimulus varies on each trial, the *average* recency remains constant across sequential segments, so the impact of forgetting remains constant. As learning increases, but forgetting stays constant, performance improves. In the Experimental condition, however, the average delay between presentations *increases* across power-law segments (Fig. 3A), while repetitions accumulate at a similar rate. Here, increased learning across power-law segments can be offset or overwhelmed by the increased forgetting.

To assess this explanation, we first aimed to fit an exemplar-forgetting model to Experiment 1. We acknowledge Devraj et al. fit an exemplar model with forgetting (Donkin & Nosofsky, 2012) and did not see an advantage over their base exemplar model. However, this may be due to their fitting method aggregated across all trials in a trial segment, which collapses across items that vary in their recency. Exemplar-forgetting instead suggests that items with different recency should vary in performance: when an item is shown twice in succession, performance should be much higher if the delay is shorter. As a result, the *average* response across trials with varied recency may be equally well captured by models with or without forgetting. We expect that by fitting models to individual trials (see *Methods*), we should see an advantage for including forgetting.

To demonstrate that an exemplar-forgetting model can also *predict* the seemingly opposed patterns of performance in both conditions with minimal assumptions, we simulated data from the exemplar-forgetting model with a single representative set of parameters. We then fit this simulated data using the base exemplar and prototype models and demonstrate that the trends of model-fit observed in Devraj et al. Experiment 1 can plausibly be generated by an exemplar-forgetting mechanism. Finally, we also formalised the representation-shift as a mixture model to directly compare with an exemplar-forgetting model.

## Model fitting

We added a power-law forgetting function (Donkin & Nosofsky, 2012) to our base exemplar model, which served to reduce the strength of exemplars relative to the delay since they were last seen. Each exemplar was multiplied by its memory strength *M* before summation resulting in the probability of categorising stimulus *i* into category A being defined as:

9 $$\mathrm{P}\left(\mathrm{A}|i\right)=\frac{\sum_{\mathrm{j}\in \mathrm{A}}{{M}_{\mathrm{j}}s}_{\mathrm{ij}}}{\sum_{\mathrm{j}\in \mathrm{A}}{{M}_{\mathrm{j}}s}_{\mathrm{ij}} + \sum_{\mathrm{j}\in \mathrm{B}}{{M}_{\mathrm{j}}s}_{\mathrm{ij}}}$$

where *M* is:

10 $${M}_{j}={j}^{-\beta }$$

with *j* indicating the delay (in trials) and $\beta$ the rate of memory decay. This raised the number of free parameters from six to seven.

Our model simulations used a procedure similar to that used by Devraj et al. (Appendix E), with a single representative set of parameters used to generated simulated responses using the stimuli sequences presented to participants. We used equal attention weights of one-sixth, with the sensitivity parameter ranging from [5.5, 10.5] increasing by.5 per-segment (sequential or power-law) to capture learning. Forgetting rate was chosen as *β* =.7. These values were used for both conditions and were chosen semi-arbitrarily to simplify our demonstration.^5^ From the predicted probabilities, we simulated a set of Bernoulli samples for all of the sequences of stimuli seen by participants. Hence, the simulated data reflect what we should expect to see if the exemplar-forgetting model is the ‘ground truth’. We ask whether the observed patterns of performance obtained from this simulated data, and the subsequent fits of exemplar and prototype models to this data, are consistent with Devraj et al. If so, exemplar-forgetting is a sufficient explanation of their phenomena.

### Exemplar-forgetting fits to Experimental data

**Fig. 6:**
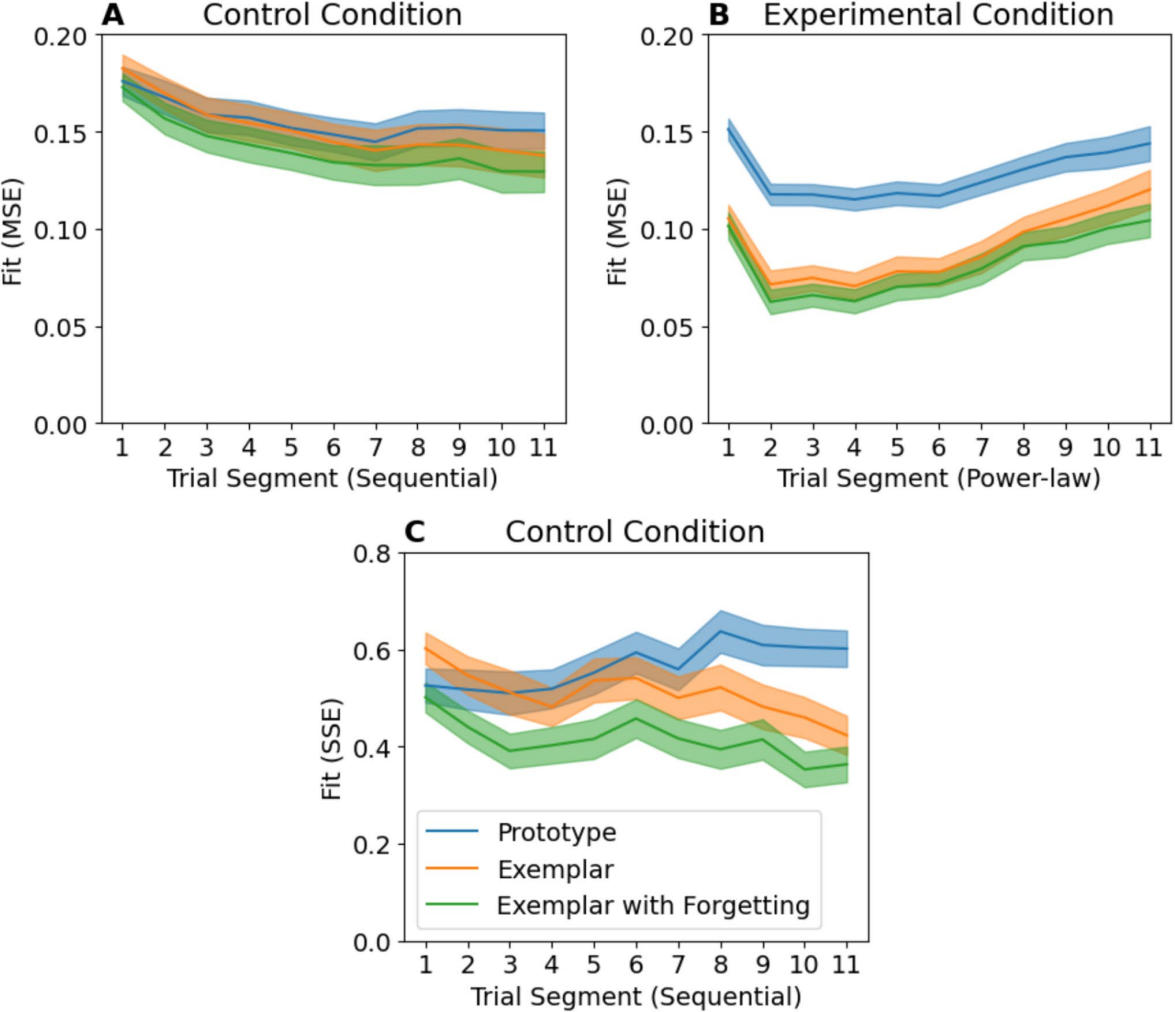
Model fit comparison between exemplar model with forgetting and the two base models using mean-squared error (MSE) in both conditions (**A, B**) and additionally using sum-squared error (SSE) in the control condition (**C**). Error bars indicate standard error of the mean

In contrast with Devraj et al., our exemplar model with forgetting showed improved fit in both conditions compared with both the base exemplar and prototype models (Fig. 6A, B). This is consistent with our hypothesis that forgetting occurred in both conditions, with recent items showing higher memory strength regardless of the stimulus distribution. This advantage was apparent regardless of fit statistic/method (Fig. 6C) and therefore was not purely the result of aggregation across trials. Instead, it may reflect Devraj et al.’s decision to select a fixed forgetting rate rather treating it as a free parameter.

### Simulated data from exemplar-forgetting model

**Fig. 7:**
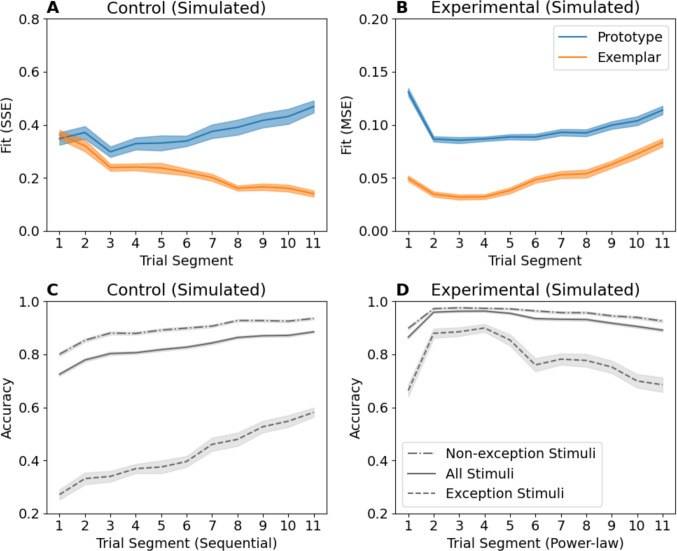
Model fits of the base prototype and exemplar models to data simulated under the exemplar-forgetting model (**A**,** B**). Note how the advantage for the exemplar model increases over time in the Control condition, and starts at its maximum before decreasing over time in the Experimental condition similarly to the fits to participant data in Fig. 4A and B. Performance shows a similar pattern, increasing over time in the Control condition (**C**), and increasing then decreasing over time in the Experimental condition (**D**), as in Fig. 4C and D

Data simulated from the exemplar-forgetting model show that the broad pattern of increasing performance across sequential segments and decreasing performance across experimental segments is *predicted* by the model (see Fig. 7C, D). The key point here is that under the same set of parameters, using a single model, what appear initially to be vastly different patterns of behaviour can be understood as similar patterns of behaviour in dissimilar designs.

When fitting the simulated data with both the exemplar and prototype models, we are also able to replicate the general qualitative trends seen in the genuine data. In the Control condition (Fig. 7A), the base exemplar fit steadily improves across sequential segments compared with the prototype fit. In the Experimental condition (Fig. 7B), this trend reverses and base exemplar fit worsens over time relative to prototype fit as performance decreases. Rather than evidence for a shift toward prototype representation, our simulation shows that a decreasing exemplar fit advantage over time can arise even with an exemplar-forgetting model producing the data. As highlighted by Devraj et al., predictions from the exemplar model are sensitive to the total number of exemplars seen, which varies systematically across power-law segments. To ensure our simulations were indeed capturing a pattern of forgetting behaviour, we investigated performance after all stimuli were encountered in Appendix A (Online Supplementary Material) and found that these trends persist.

### Mixture model

Finally, we tested how well a ‘representation shift’ explanation compares to the exemplar-forgetting model. While ideally the ‘representation shift’ model would define how category representations are combined, implementing any specific mechanism risks misrepresenting Devraj et al.’s claims. Instead, we implemented a simple mixture model consisting of a weighted combination of both prototype and exemplar model predictions where the probability of a category A response given stimulus *i* is given by:

8 $$P\left(\mathrm{A}|i\right)= \alpha \cdot {P\left(\mathrm{A}|i\right)}_{\mathrm{prototype}}+\left(1-\alpha \right)\cdot {P\left(\mathrm{A}|i\right)}_{\mathrm{exemplar}}$$

where $\alpha$ is the mixture parameter. Each component model had separate sensitivity parameters^6^ for a total of eight free parameters in the Control condition compared with seven for the exemplar-forgetting model. As with previous analyses, all parameters varied by sequential segment in the Control condition. The mixture parameter was allowed to vary across power-law segments in the Experimental condition (other models shared all parameters across segments). While this makes the mixture model particularly complex in the Experimental condition, mixture parameter variation across segments could potentially be simplified with an appropriate linking function.^7^

**Fig. 8:**
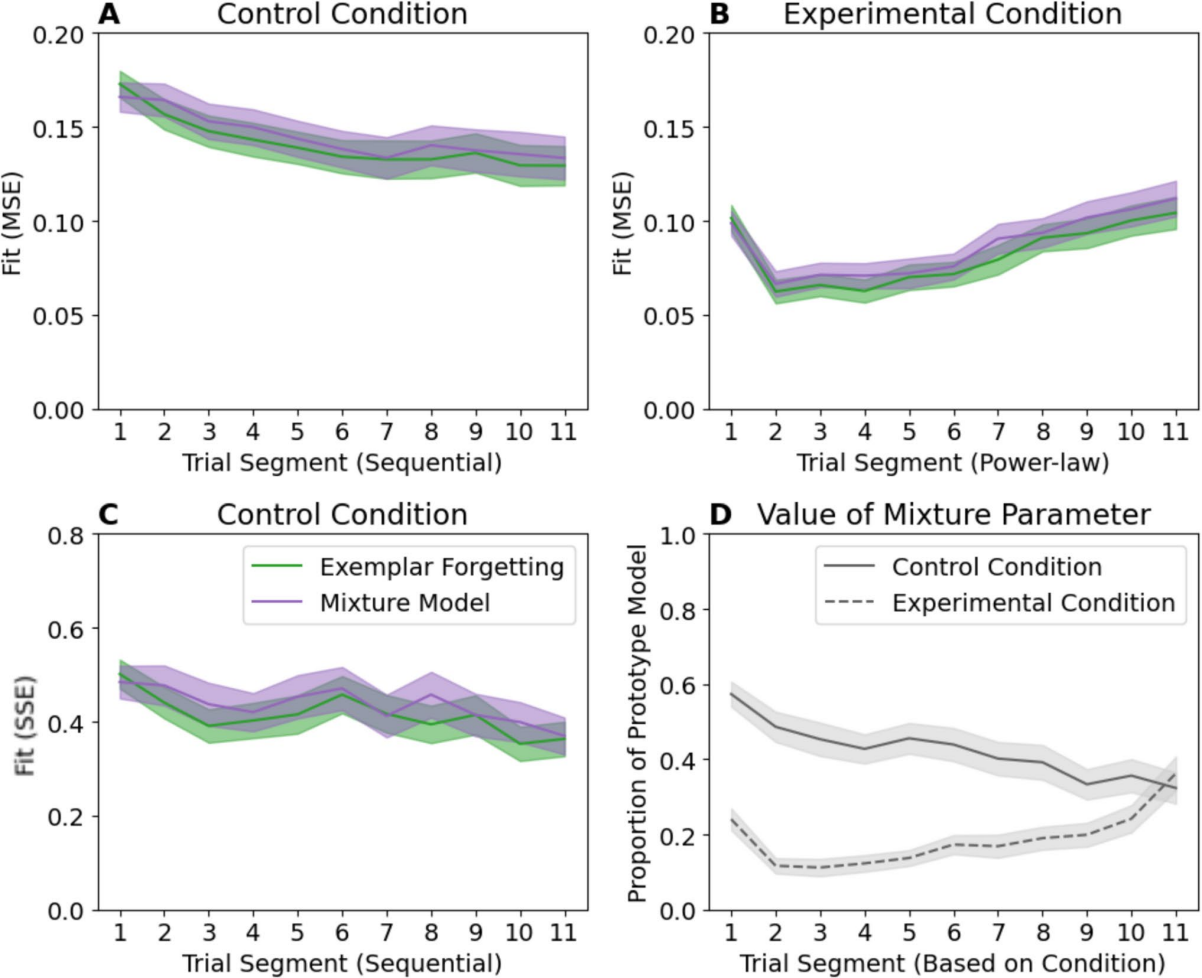
Fits of the exemplar-forgetting and mixture models to the Control and Experimental conditions using mean-squared error (MSE) (**A**, **B**) and sum-squared error (SSE) to the control condition (**C**). Values of the mixture parameter across segments (**D**) are from the MSE fits

Consistent with the representation-shift described by Devraj et al., the estimated mixture parameter (see Fig. 8D) suggested a decrease in prototype model use over time in the Control condition, and increased use over time in the Experimental condition. However, mixture fit was at best equivalent to the exemplar-forgetting model (Fig. 8A, B and C) in both conditions despite increased complexity. As a simplified mixture model remains plausible, we suggest that the current data are not adequately diagnostic to definitively rule out either explanation through model fit alone.

## General discussion

In summary, Devraj et al. argue that participants shift reliance on representational strategies in response to memory constraints imposed by the stimulus distribution. Specifically, when stimuli are presented randomly, all items are equally important regardless of recency. Participants respond by maintaining old exemplars (rather than forgetting them), encouraging exemplar-based classification strategies, and leading to improved performance over time through associative learning. Alternatively, when presented according to a power-law distribution, recent stimuli are more likely to be task-relevant and are preferentially maintained. This impairs access to exemplar memories over time, reducing performance and resulting in a reliance on prototype representations. In line with the advantage of an exemplar model with a forgetting function, we instead argue that power-law forgetting underlies performance in both conditions. When stimuli are distributed randomly, the average delay between exemplar presentations, and thus the effect of forgetting, remains constant over time. However, in the power-law experimental condition, delay increased across segments, which consequently decreased both exemplar strength and performance. Although the control and experimental conditions saw opposing trends of growing and declining exemplar model-fit advantage, our model simulations demonstrated that these seemingly opposed patterns are predicted a priori by an exemplar-forgetting model. Rather than reflecting changes in the employment of prototype and exemplar category representations, they instead reflect changes in how diagnostic the data are for distinguishing the two representations. While both the exemplar-forgetting and strategic-shift accounts were equally capable of fitting the data when formalised as computational models, we argue that an exemplar-only account is more parsimonious.

Where prototype and exemplar models assume that categories are represented in memory by either prototypes or exemplars, respectively, the strategic-shift account requires that both are stored. It may seem that only an exemplar store is required, as category prototypes could easily be constructed from retrieved exemplars. However, if exemplars remain accessible over time, abstracting a prototype is an unnecessary additional computation which reduces performance. Alternatively, if exemplars become inaccessible, prototype information should also degrade. As Devraj et al. suggests, for a representation-shift to be adaptive, both stores must exist simultaneously, with the exemplar store decaying more rapidly. Although such a multiple-representation model is possible, we highlight that it is significantly more flexible than either single-representation alternative and thus should only be accepted if more parsimonious accounts are insufficient.

The representation-shift account additionally suggests that different patterns of performance are the result of adaptation to the stimulus distribution and only prioritising recent information when it is more informative. We highlight that inferring the relative frequencies or distribution of items necessitates knowledge of multiple copies of each stimulus and when they appeared. By contrast, exemplar-forgetting only requires a single copy of each exemplar being stored, which then decays over time. If recent information is always prioritised, this will lead to improved performance when recent information is in fact more informative without requiring any ability to track exemplar frequencies. If instead people prioritise recent information adaptively based on stimulus distribution in categorisation tasks, we question why similar behaviour is not observed in memory paradigms.

Across a diversity of memory tasks, power-law curves provide the strongest account of forgetting (Rubin & Wenzel, 1996; Wixted & Carpenter, 2007; Wixted & Ebbesen, 1991). In recognition memory specifically, Donkin and Nosofsky (2012) demonstrated that response times and accuracy are closely fit by a power-law forgetting curve despite target words being sampled uniformly from various delays – thus lacking environmental recency. Anderson and Schooler (1991) argued that power-law forgetting may have been an adaptation to the constraints of the world, where recent information has a higher need probability than earlier information. However, Anderson and Schooler did not explain *how* adaptation occurs. We argue that rather than dictating strategies on cognitive tasks, this adaptation may have influenced the *structure* of our cognitive system. Even if forgetting originated as an adaptation to environmental statistics, it appears that participants maintain recency patterns across a wide range of tasks and stimulus sets where environmental recency is no longer present. Note we are not arguing against task-adaptive strategies generally. Active rehearsal in memory tasks and rule-based categorisation are clear examples. Instead, we argue that these strategies are *responses* to immutable patterns of forgetting.

While behaviour on memory and categorization tasks can appear to conflict, often investigating the predictions of computational models can explain this as changes in task demands. We suggest that what may initially appear to be evidence for strategic adaptation based on the environmental distribution of recency can instead be interpreted as a direct consequence of systematic recency manipulation without necessitating multiple strategies. Although such strategic utilisation of both prototype and exemplar representations remains plausible, we argue that exemplar-forgetting alone can explain the present data. Future work should emphasise paradigms more suited to producing diagnostic differences between model predictions using more complex category structures, or tasks such as category generalisation.

## Supplementary Information

Below is the link to the electronic supplementary material.

Supplementary file1 (DOCX 188 KB)

## Data Availability

For the experiment code and original analysis, see the GitHub repository for the original work (https://github.com/arjundevraj/rational-categorization, https://github.com/arjundevraj/word_categorization).

## Footnotes

1 Specifically, exemplar models which allow for variation in the amount of deterministic compared with probabilistic responding do not struggle to capture these patterns (Nosofsky & Zaki, 2002).

2 This meant models were fit twice in the Control condition – once using each statistic/aggregation method.

3 Note, this is a simplified explanation – selective attention and memory strength are also relevant and discussed later.

4 This may seem counterintuitive, but consider that segments in each condition reflect different things. Sequential segments simply reflect temporal subdivisions of the experiment, whereas power-law segments reflect how far along a stimuli’s power-law distribution this trial lies.

5 To expand on this, attention weights should also likely vary with learning, and our linear increase in sensitivity is probably overly simplistic. Learning rate is set to approximately the mean value obtained across all participants, segments, and conditions.

6 We initially allowed separate attention weights for both models; however, the improvement in fit was tiny – a mean MSE improvement of.002 in both conditions, where the difference between the mixture and exemplar-forgetting model is approximately.006. Choice of either parameterisation did not alter overall conclusions about fit or complexity.

7 This would likely relate to the need-odds described by Devraj et al.; however, we do not wish to make any assumptions about what relationship would be appropriate.

## Code availability

All data and analysis code are available at our Open Science Framework (OSF) repository (https://osf.io/2mav6/).

## Declarations

### Open practices statement

Data and analysis code are available on the OSF (https://osf.io/2mav6/). For experiment code and the original analysis, see the GitHub (https://github.com/arjundevraj/rational-categorization, https://github.com/arjundevraj/word_categorization). The experiments were also pre-registered (Experiment 1: https://aspredicted.org/yq984.pdf; Experiment 2: https://aspredicted.org/9me7s.pdf).
