# Supplementary material for: Recency is sufficient for reconciling categorisation and memory: Commentary on Devraj et al. (2024)
Source: Psychon Bull Rev. 2026 Jan 15;33(1):48. doi: 10.3758/s13423-025-02780-9 (PMC12808293; doi:10.3758/s13423-025-02780-9)
Supplement: Supplementary file 1 — Supplementary file1 (DOCX 188 KB) [file 13423_2025_2780_MOESM1_ESM.docx]

**Appendix**

**A. Investigation of power-law Experimental condition confound in simulated data**

As discussed in Appendix E of Devraj et al. (2024), the Experimental condition contains a confound when interpreting both classification accuracy and model fits. As stimuli were introduced one-by-one, participants experienced fewer unique exemplars early in the experiment. Also, until all exemplars were introduced, they often saw more exemplars from one category than another and may not have encountered any exception items. This does not impact prototype model predictions, however exemplar models predict that categories containing more exemplars have higher similarity to any given item. Although power-law segments grouped stimuli by frequency rather than chronologically, because stimuli always initially appearing at high frequency early power-law segments contain proportionally more trials from earlier in the experiment. As a result, trials in early power-law segments correspond with trials where participants had encountered few unique stimuli whereas trials in late segments usually reflect participants who have seen most unique exemplars. When accuracy and exemplar-model fit declines across power-law segments, some of this may be a result of this confound rather than an effect of the power-law manipulation.

Devraj et al. addressed this confound in Appendix E. by analysing simulated fits from both models for trials after which all stimuli were encountered (approximately 25% of the data). They found that MSE fit to simulated data for both models was flat across trial segments when only these trials were included. We suggest that simulated fits are challenging to interpret in isolation, so instead began by examining both observed and simulated classification accuracy in this subset of trials in Figure 9 below.

We found that, mirroring the trends seen in the non-filtered data, accuracy declined across power-law segments as the delay between exemplar repetitions increased (Figure 9A). As expected, data simulated from the exemplar-forgetting model (Figure 9C) mirrored this pattern, however the base exemplar model (Figure 9B) predicted no decline in performance. This demonstrates both that this
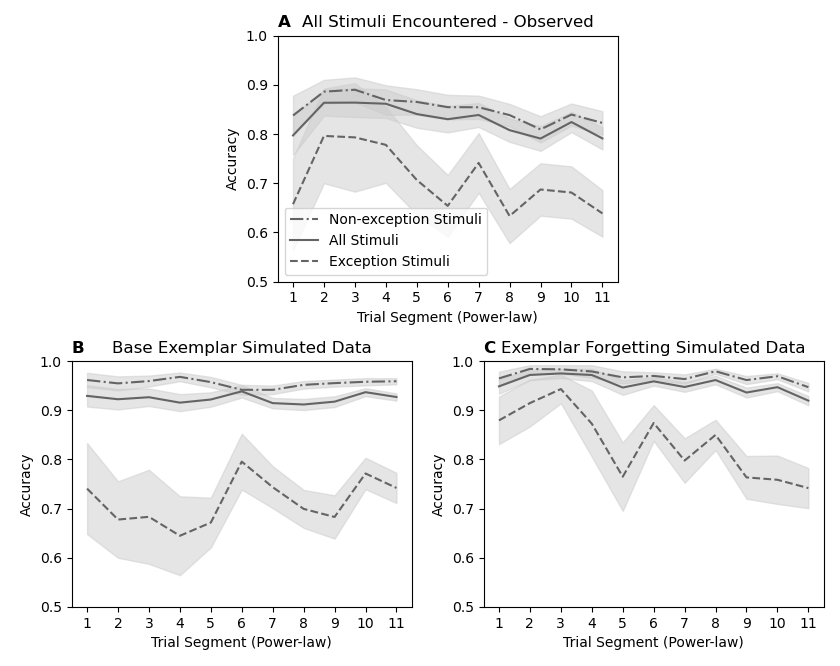


*Figure 9*. Observed (**A**) and simulated (**B, C**) classification accuracy in the Experimental condition, filtered to include only trials after all unique exemplars had been encountered by the participant.

confound alone cannot explain the decline in classification accuracy, and that the exemplar-forgetting model predicts this decline as the result of increased forgetting rather than due to the confound.

We then compared base prototype and exemplar fits to this subset of Experimental condition trials and found that the patterns of fit to observed data was replicated by fits to the simulated data (Figure 10). This suggests that the decrease in exemplar model advantage across power-law segments is not merely the result of an increase in the number of stimuli analysed and instead is the result of the observed decline in performance.


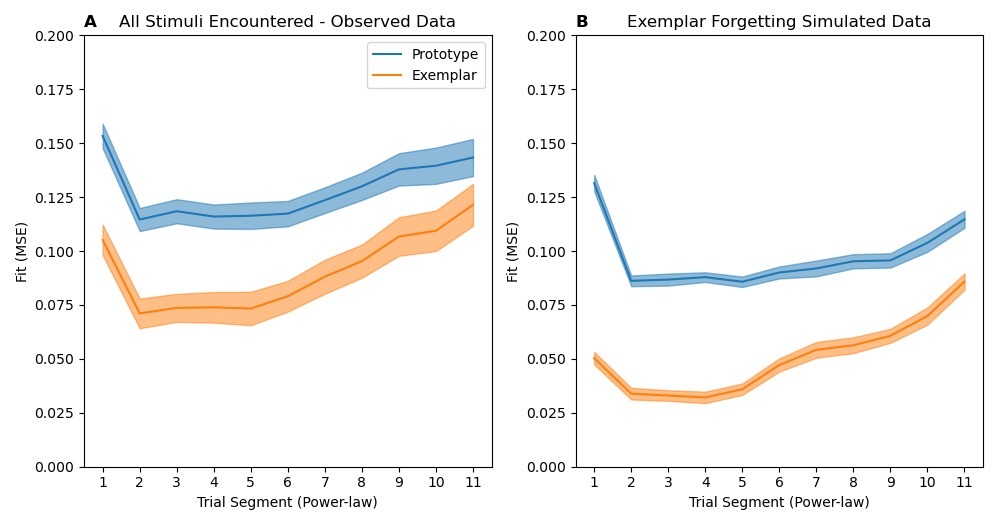


*Figure 10.* Model fits of the base prototype and exemplar models to the subset of Experimental condition trials where all stimuli had been encountered (**A**) and the same subset of trials simulated from the exemplar-forgetting model (**B**).
